# Reactivating TP53 signaling by the novel MDM2 inhibitor DS-3032b as a therapeutic option for high-risk neuroblastoma

**DOI:** 10.18632/oncotarget.23409

**Published:** 2017-12-18

**Authors:** Viktor Arnhold, Karin Schmelz, Jutta Proba, Annika Winkler, Jasmin Wünschel, Joern Toedling, Hedwig E. Deubzer, Annette Künkele, Angelika Eggert, Johannes H. Schulte, Patrick Hundsdoerfer

**Affiliations:** ^1^ Charité - Universitätsmedizin Berlin, Corporate Member of Freie Universität Berlin, Humboldt-Universität zu Berlin, and Berlin Institute of Health, Department of Pediatric Hematology/Oncology/Stem Cell Transplantation, Berlin, Germany; ^2^ Berlin Institute of Health (BIH), Anna-Louisa-Karsch 2, Berlin, Germany; ^3^ Neuroblastoma Research Group, Experimental and Clinical Research Center (ECRC), Berlin, Germany; ^4^ German Cancer Consortium (DKTK), Heidelberg, Germany; ^5^ German Cancer Research Center (DKFZ), Heidelberg, Germany

**Keywords:** apoptosis, CDKN1A, MYCN, pediatric tumors, targeted therapy

## Abstract

Fewer than 50% of patients with high-risk neuroblastoma survive five years after diagnosis with current treatment protocols. Molecular targeted therapies are expected to improve survival. Although MDM2 has been validated as a promising target in preclinical models, no MDM2 inhibitors have yet entered clinical trials for neuroblastoma patients. Toxic side effects, poor bioavailability and low efficacy of the available MDM2 inhibitors that have entered phase I/II trials drive the development of novel MDM2 inhibitors with an improved risk-benefit profile. We investigated the effect of the novel MDM2 small molecular inhibitor, DS-3032b, on viability, proliferation, senescence, migration, cell cycle arrest and apoptosis in a panel of six neuroblastoma cell lines with different *TP53* and *MYCN* genetic backgrounds, and assessed efficacy in a murine subcutaneous model for high-risk neuroblastoma. Re-analysis of existing expression data from 476 primary neuroblastomas showed that high-level *MDM2* expression correlated with poor patient survival. DS-3032b treatment enhanced TP53 target gene expression and induced G1 cell cycle arrest, senescence and apoptosis. CRISPR-mediated *MDM2* knockout in neuroblastoma cells mimicked DS-3032b treatment. TP53 signaling was selectively activated by DS-3032b in neuroblastoma cells with wildtype *TP53*, regardless of the presence of *MYCN* amplification, but was significantly reduced by *TP53* mutations or expression of a dominant-negative TP53 mutant. Oral DS-3032b administration inhibited xenograft tumor growth and prolonged mouse survival. Our *in vitro* and *in vivo* data demonstrate that DS-3032b reactivates TP53 signaling even in the presence of *MYCN* amplification in neuroblastoma cells, to reduce proliferative capacity and cause cytotoxicity.

## INTRODUCTION

Inactivation of the crucial tumor suppressor, TP53, is a common event in tumorigenesis. TP53 activity can be functionally inhibited by *TP53* mutation or deregulating components of the TP53 pathway. Next-generation sequencing in 32 cancer types established that *TP53* mutations occur in 35% of cancers [1]. However, in neuroblastoma, the most common extracranial solid tumor of childhood, fewer than 2% of primary neuroblastomas [2–4] and 14% of relapsed neuroblastomas [5] harbor *TP53* mutations. Deregulating MDM2 proto-oncogene expression is one effective mechanism to impede TP53 activity. MDM2-TP53 binding is known to inhibit TP53 transcriptional activity [6]. MDM2 also has E3 ubiquitin ligase activity that has been demonstrated to cause polyubiquitination of TP53, leading to proteasomal degradation [7]. *MDM2* itself is a transcriptional TP53 target, indicating the presence of a negative autoregulatory feedback loop between MDM2 and TP53 [8]. Aberrant MDM2 activation has been suggested as a possible mechanism by which neuroblastoma cells escape death. In a study of 41 primary tumors, 36.6% harbored either an *MDM2* amplification or a mutational or epigenetic inactivation of *CDKN2A*, a negative regulator of MDM2 [5]. *MYCN* amplification occurs in approximately 45% of primary high-risk neuroblastomas and is the strongest independent negative prognostic risk factor in patients [9]. *MDM2* and *TP53* are MYCN transcriptional targets [10, 11], and MDM2 is a translational regulator of *MYCN* via mRNA stabilization in the cytoplasm [12]. MDM2 haploinsufficiency inhibits tumor formation in a MYCN-driven neuroblastoma mouse model [13]. Despite the low mutation rate of *TP53* in neuroblastoma, the TP53-MDM2 axis appears to be deregulated in at least a subgroup of high-risk neuroblastomas, identifying it as an actionable target.

The possibility to reactivate TP53 signaling by modulating MDM2-TP53 activity drove design and development of several small molecule inhibitors over the last 13 years. Nutlin-3 was the first selective MDM2 inhibitor shown to activate TP53 and downstream signaling in preclinical neuroblastoma models [14–17]. Several other chemical classes of MDM2 inhibitors have been developed, among which RG7112, RG7388, MI-63, NDD0005 and MI-773 have been demonstrated to suppress neuroblastoma cell viability and proliferation in preclinical models [18–23]. None of these inhibitors has proceeded to clinical trials with neuroblastoma patients to date. Limited *in vivo* potency and poor bioavailability have prohibited translation of the initially designed molecules into clinical trials [24, 25]. Early clinical trials with MDM2 inhibitors in adult patients were also limited by toxicity [26]. Even though several MDM2 inhibitors have already been tested in preclinical models of neuroblastoma and MDM2 validated as a promising target, the need remains to identify, develop and preclinically assess novel MDM2 inhibitors with greater efficacy, improved bioavailability and fewer toxic side effects.

Despite aggressive multimodal treatment strategies, long-term survival remains below 50% in patients with high-risk neuroblastoma [27], and outcome for patients with relapsed neuroblastoma is almost always fatal [28, 29]. Molecular targeted therapies such as MDM2 inhibitors are expected to improve patient outcome. DS-3032b is a novel orally available, dispiropyrrolidine-based compound that impairs MDM2 binding to the TP53 transcriptional activation domain. To date, preclinical testing of DS-3032b has not been reported. Initial results emerging from a phase I trial (NCT02319369) treating adults with relapsed/refractory hematological malignancies have shown that DS-3032b has pharmacodynamic activity and shows evidence of clinical efficacy (reduction of blast cells in bone marrow following 15 cycles in 15 of 26 patients) with acceptable clinical side effects that included myelosuppression, nephrological and gastrointestinal symptoms [30]. Two further phase I trials are currently evaluating DS-3032b as a single agent in adult patients with advanced solid tumors or lymphomas (NCT01877382) or with relapsed/refractory multiple myeloma (NCT02579824), but it is too early to draw any conclusions. Given the growing clinical experience with DS-3032b in adults, it is well poised to enter trials for pediatric patients with cancers against which preclinical efficacy can be demonstrated.

We preclinically evaluated the potential of DS-3032b for high-risk neuroblastoma. Neuroblastoma cell lines and xenograft tumor models were used to test efficacy and characterize the mechanisms of DS-3032b action resulting in TP53-mediated induction of cell cycle arrest, apoptosis and senescence. Our aim is to provide preclinical data to support the incorporation of DS-3032b into trials applying combination treatment regimens that include molecular targeted inhibitors for patients with high-risk, primary refractory or relapsed neuroblastoma.

## RESULTS

### High-level *MDM2* expression in neuroblastoma predicts poor patient survival

**Figure 1:**
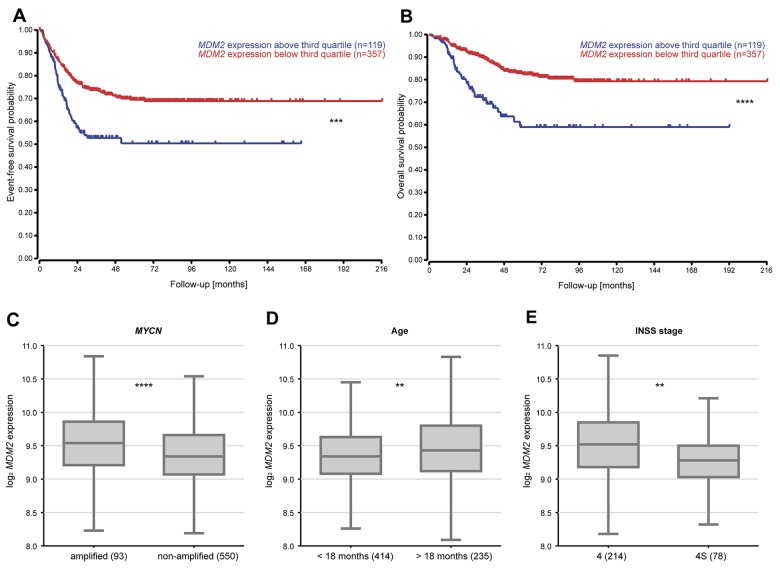
Elevated *MDM2* expression levels in tumors are associated with poor patient overall and event-free survival as well as more aggressive neuroblastomas **(A-B)** Tumors from a cohort of 476 primary tumors were classified into high- or low-expressing groups according to whether *MDM2* expression was greater or lower than the third quartile *MDM2* expression. Kaplan–Meier analysis of overall and event-free patient survival. **(C-E)** Association of *MDM2* expression with prognostic markers (*MYCN* status, age, INSS stage) in 649 primary tumors. ^**^ = P < 0.01, ^***^ = P < 0.001, ^****^ = P < 0.0001.

TP53 inactivation via mutation is known to occur in less than 2% of primary neuroblastomas [2–4], but TP53 function can also be disrupted by deregulated *MDM2* expression. We reanalyzed microarray expression data from a cohort of 476 primary neuroblastomas [31] to assess correlations between *MDM2* expression and patient prognosis. We used *MDM2* expression in the tumors to segregate patients into the upper quartile and the lower three quartiles. Kaplan–Meier analysis showed that high-level *MDM2* expression in tumors correlated with poor event-free and overall survival (Figure 1A-1B). Within stage 4 neuroblastoma, correlation between MDM2 expression and poor event-free and overall survival is present in *MYCN* non-amplified tumors, but not in *MYCN* amplified tumors (Supplementary Figure 1). Three known risk factors, the presence of a *MYCN* amplification in the tumor, patient age > 18 months at diagnosis and stage 4 disease according to the International Neuroblastoma Staging System (INSS), significantly correlated with elevated *MDM2* expression in tumors (Figure 1C-1E). These results indicate that elevated *MDM2* expression in neuroblastomas is associated with more aggressive disease. For this reason, patients with high-risk neuroblastoma may benefit from a therapy targeting TP53-MDM2 signaling.

### DS-3032b inhibits neuroblastoma cell growth and migration, and induces cell cycle arrest, senescence and apoptosis in a functional TP53 background

**Figure 2:**
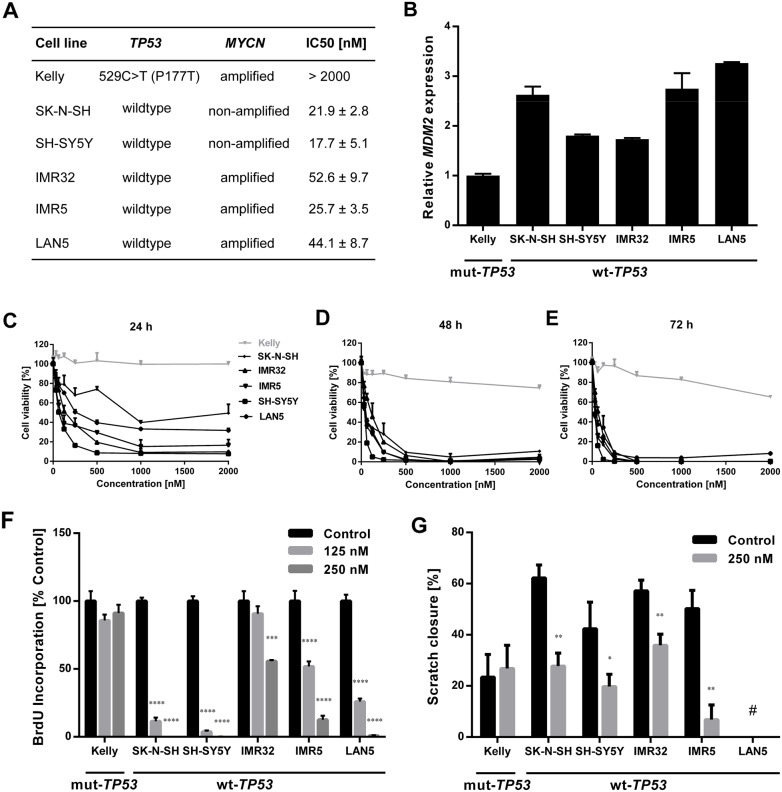
DS-3032b treatment selectively inhibits viability, proliferation and migration of neuroblastoma cells with wildtype *TP53* independently of *MYCN* status **(A)** *TP53* mutational status, *MYCN* status and IC50 of DS-3032b after 72 h exposure in neuroblastoma cell lines. **(B)** *MDM2* expression was higher in neuroblastoma cells with wildtype *TP53* in comparison to the *TP53* mutant cell line Kelly. **(C-E)** Dose-response curves for neuroblastoma cell lines treated with DS-3032b for 24, 48 and 72 h. **(F** and **G)** Proliferation (measured by BrdU ELISA) and migration (measured by scratch assay) were reduced by treatment with DS-3032b in cell lines with wildtype *TP53*, whereas the *TP53* mutant cell line Kelly remained unaffected. Data represent mean values and SD (n = 3 per group). ^*^ = P < 0.05, ^**^ = P < 0.01, ^***^ = P < 0.001, ^****^ = P < 0.0001. ^#^ = scratch assay was technically unfeasible for LAN5 cells due to poor adherence to cell culture plastic.

We assessed the effect of the novel small molecule MDM2 inhibitor, DS-3032b, on several hallmarks of cancer in a panel of six neuroblastoma cell lines with varying genetic backgrounds. Two cell lines (SK-N-SH, SH-SY5Y) were included that lacked *MYCN* amplifications as controls for the diploid *MYCN* genetic background. The other four cell lines harbored *MYCN* amplifications. The selected neuroblastoma cell lines were also reported to harbor wildtype or mutant *TP53*. We validated the *TP53* mutational status in all six cell lines by sequence analysis of the entire *TP53* coding region (Figure 2A). Our analyses confirmed published reports, and showed that SK-N-SH, SH-SY5Y, IMR32, IMR5 and LAN5 cell lines harbor wildtype *TP53*, while the Kelly cell line harbors a 529C>T (P177T) missense mutation in *TP53*. Relative *MDM2* expression was higher in the five neuroblastoma cell lines lacking *TP53* mutations than in Kelly cells (Figure 2B). DS-3032b treatment reduced viability in the SK-N-SH, SH-SY5Y, IMR32, IMR5 and LAN5 cell lines in a dose- and time-dependent manner, while the only cell line harboring a *TP53* mutation (Kelly) was remarkably less sensitive to DS-3032b treatment (Figure 2C-2E). We directly examined the effect of DS-3032b on proliferation using a bromodeoxyuridine (BrdU) enzyme-linked immunosorbent assay (ELISA) assay. DS-3032b treatment for 48 h also reduced BrdU incorporation in all cell lines except Kelly in a dose-dependent manner (Figure 2F). The effect of DS-3032b treatment on cell migration was investigated using the scratch assay in all cell lines except LAN5, which does not adhere strongly to cell culture plastic making this assay technically infeasible. Scratch healing was reduced in all cell lines except Kelly by 24-hour treatment with 250 nM DS-3032b (Figure 2G). The novel MDM2 inhibitor, DS-3032b, suppressed viability, proliferation and migratory capacity in neuroblastoma cell lines with functional TP53, regardless of the presence of *MYCN* amplifications.

**Figure 3:**
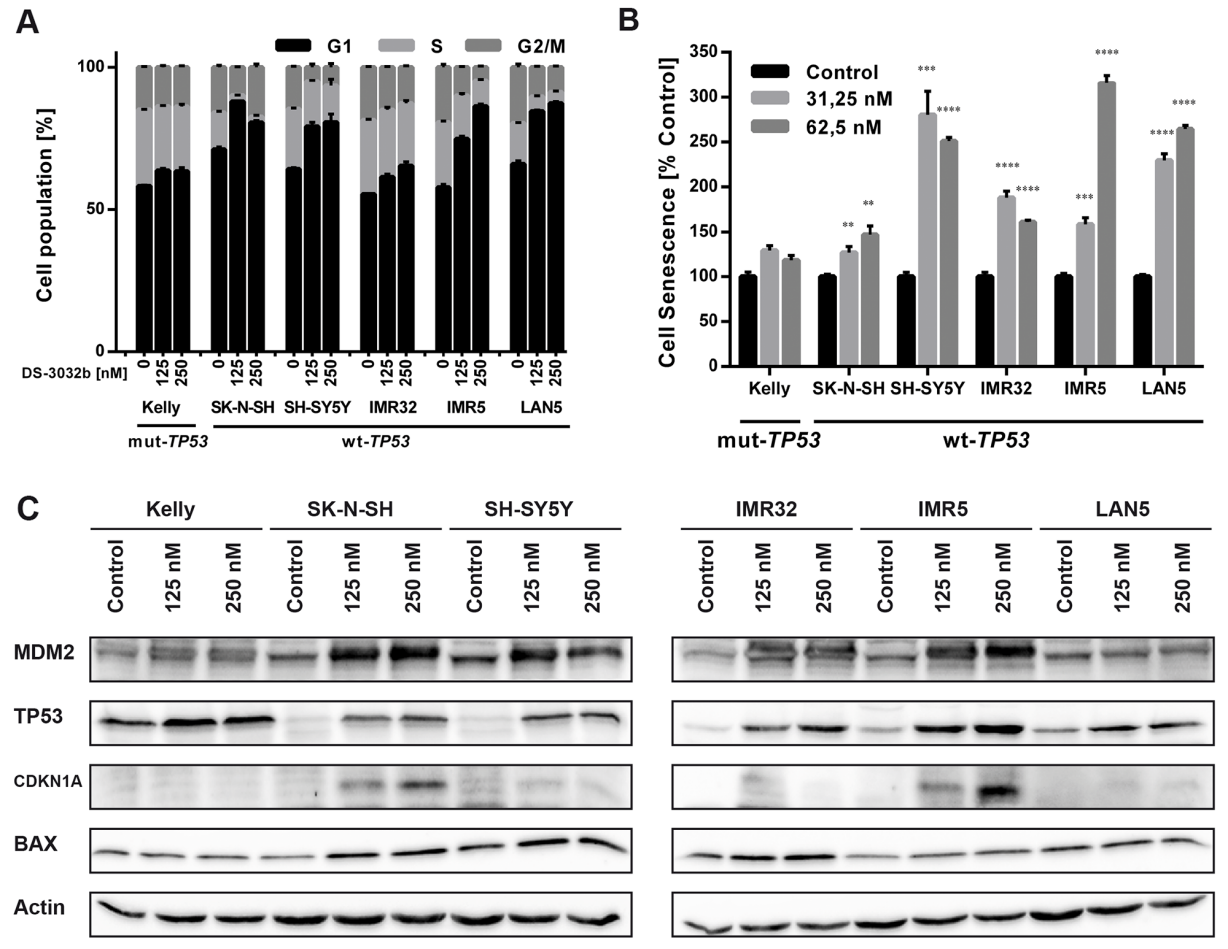
DS-3032b treatment stabilizes TP53 and selectively induces expression of TP53 target genes in neuroblastoma cells with wildtype *TP53* leading to G1 cell cycle arrest and senescence induction **(A)** DS-3032b treatment induced accumulation of cells with wildtype *TP53* in G1 phase and a decrease of cells in S phase in a dose-dependent manner, but not in Kelly cells. **(B)** Senescence (measured by senescence-associated beta-galactosidase (SA-β-gal) activity assay) was induced by DS-3032b treatment in cell lines with wildtype *TP53*, whereas Kelly cells remained unaffected. **(C)** Activation of the TP53 signaling was shown by induction of MDM2, CDKN1A and BAX in neuroblastoma cells harboring wildtype *TP53*, in comparison to Kelly cells. Data represent mean values and SD (n = 3 per group). ^**^ = P < 0.01, ^***^ = P < 0.001, ^****^ = P < 0.0001.

**Figure 4:**
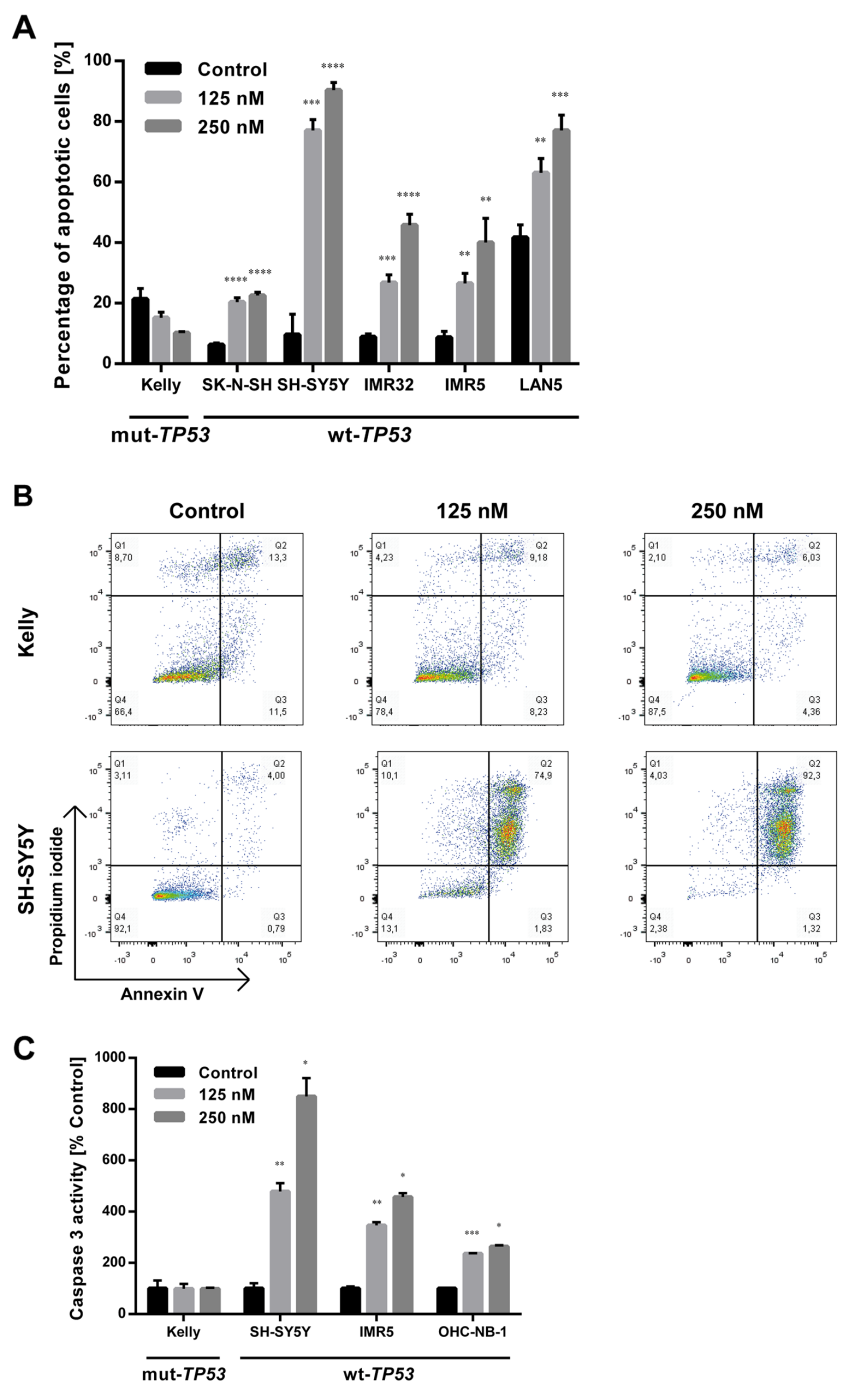
DS-3032b treatment selectively induces apoptosis in neuroblastoma cells with wildtype *TP53* **(A)** Fraction of early (annexin V positive, propidium iodide (PI) negative) and late apoptotic cells (annexin V and PI positive) was increased by DS-3032b exposure in a dose-dependent manner in neuroblastoma cell lines harboring wildtype *TP53*, in contrast to the *TP53* mutant cell line Kelly. **(B)** Exemplary dot-plots of the neuroblastoma cell lines Kelly and SH-SY5Y after DS-3032b treatment showing an induction of double-positive cells (annexin V and PI positive) in cells with wildtype *TP53*, but not in cells with mutated *TP53*. **(C)** DS-3032b exposure increased caspase 3 activity in neuroblastoma cell lines (SH-SY5Y and IMR5) and primary neuroblastoma cells (OHC-NB-1) that harbor wildtype *TP53*, in contrast to Kelly cells. Data represent mean values and SD (n = 3 per group). ^*^ = P < 0.05, ^**^ = P < 0.01, ^***^ = P < 0.001, ^****^ = P < 0.0001.

**Figure 5:**
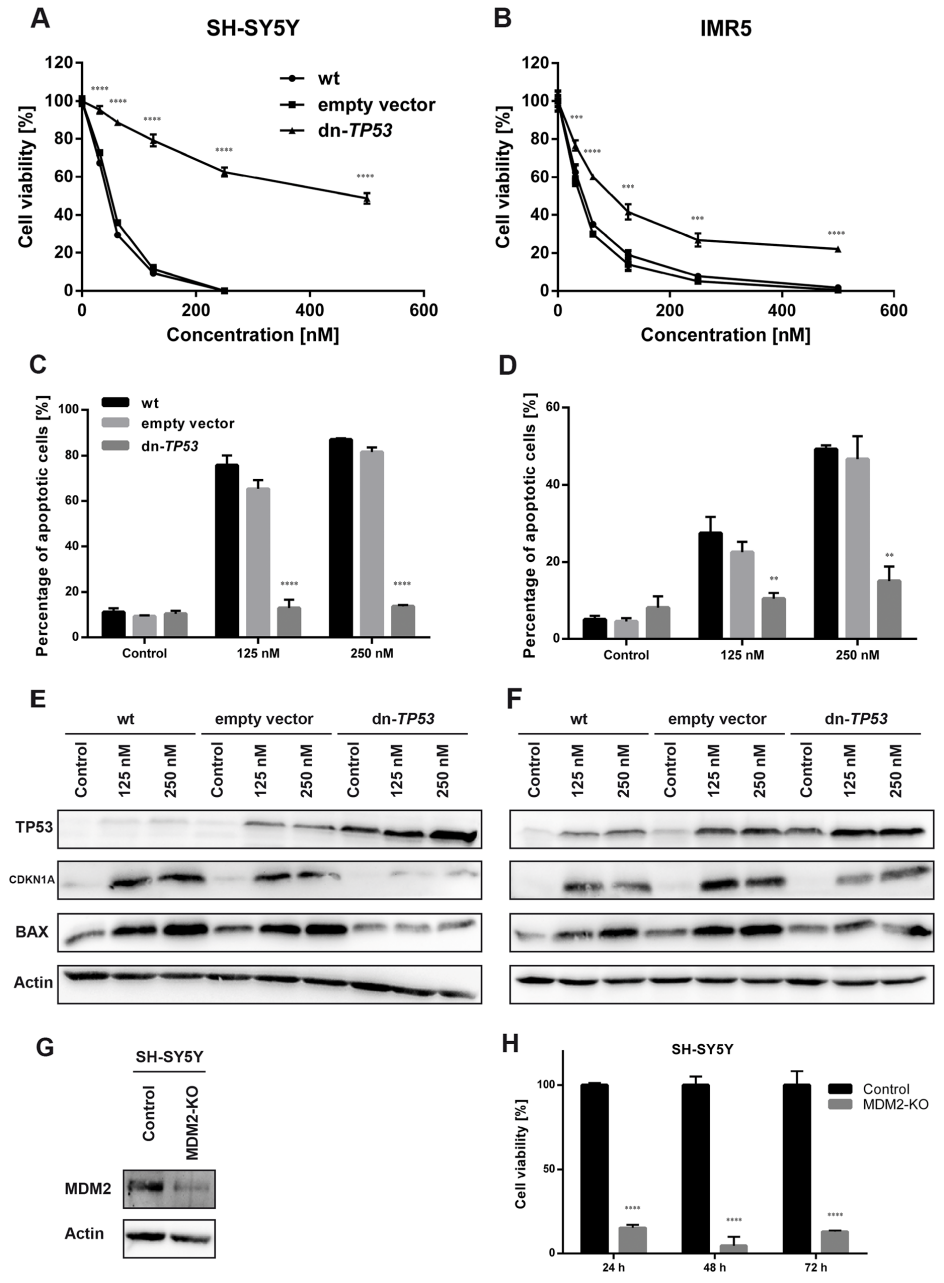
Stable, ectopic expression of dominant-negative TP53 (dn-TP53) attenuates antiproliferative and pro-apoptotic activity of DS-3032b, and CRISPR-mediated *MDM2* knockout (KO) mimics DS-3032b treatment **(A-D)** Transfection with a plasmid encoding dn-TP53 rescued SH-SY5Y and IMR5 cells from DS-3032b mediated viability inhibition and apoptosis induction. **(E-F)** Basal level of TP53 in SH-SY5Y and IMR5 cells were increased by dn-TP53 transfection, but not by transfection with an empty control vector. DS-3032b mediated induction of CDKN1A and BAX expression was attenuated in dn-TP53 expressing SH-SY5Y and IMR5 cells. **(G-H)** *MDM2* knockout was performed using CRISPR vectors (GFP-tagged) and resulted in cell viability inhibition of SH-SY5Y cells. Fluorescence activated cell sorting for GFP-expressing cells was done 24 h after transfection. Cell viability (XTT assay) was measured 24, 48 and 72 h after sorting, and cells were harvested for western blotting 7 days after sorting. Data represent mean values and SD (n = 3 per group). ^**^ = P < 0.01, ^***^ = P < 0.001, ^****^ = P < 0.0001.

DS-3032b antiproliferative activity should result from the reactivation of TP53 target gene transcriptional activation, since it prevents MDM2 binding to the TP53 transactivation domain. MDM2 inhibition has been shown to induce cell cycle arrest, senescence or apoptosis in neuroblastoma cells [15, 16, 22, 23]. To test this hypothesis for DS-3032b, we assessed the effect of DS-3032b on these TP53-dependent cellular responses. Flow cytometric analysis produced cell cycle distribution profiles following 48 h of treatment with 125 and 250 nM. DS-3032b treatment of neuroblastoma cell lines with wildtype *TP53* enriched the proportion of cells in G1 and diminished the proportion of cells in S in a dose-dependent manner (Figure 3A), indicating that DS-3032b induces arrest at the G1/S transition. DS-3032b treatment did not alter the cell cycle profiles in Kelly cells (Figure 3A), confirming that functional TP53 is necessary to arrest cells at the G1/S transition. We also measured a dose-dependent increase in cell senescence in all cell lines with wild-type *TP53* using a quantitative reporter assay that colorimetrically measures senescence-associated beta-galactosidase (SA-β-gal) activity (Figure 3B). We investigated the impact of DS-3032b treatment on the expression of TP53 and its downstream targets, MDM2, CDKN1A and BAX, using western blotting. DS-3032b upregulated MDM2, CDKN1A and BAX expression only in cell lines harboring wildtype *TP53* (Figure 3C). Treatment strongly increased TP53 expression in cell lines harboring wildtype *TP53*, and slightly increased TP53 expression in Kelly cells (Figure 3C). The moderate TP53 accumulation in Kelly cells is compatible with an increased stabilization of TP53 caused by the release from MDM2-mediated degradation. DS-3032b treatment induced apoptosis in all cell lines harboring wildtype *TP53* in a dose-dependent manner in experiments flow cytometrically detecting annexin V and propidium iodide (PI) staining (Figure 4A-4B) and caspase 3 activity (Figure 4C). To add further preclinical evidence for DS-3032b activity in neuroblastoma, we demonstrated that DS-3032b treatment induced apoptosis in primary neuroblastoma cells (OHC-NB-1) by measuring caspase 3 activity (Figure 4C). DS-3032b treatment did not influence apoptosis in the Kelly cell line. We used ectopic expression of a dominant-negative TP53 mutant (dn-TP53) to evaluate whether the effects of DS-3032b in neuroblastoma cells are a direct consequence of wildtype TP53 activation. Stable, ectopic dn-TP53 expression severely attenuated the DS-3032b-mediated reduction in viability, induction of apoptosis and increase in endogenous TP53 levels in both SH-SY5Y and IMR5 cells (Figure 5A-5F). Expression of dn-TP53 also attenuated the increase in CDKN1A and BAX expression in SH-SY5Y and IMR5 cells treated with DS-3032b (Figure 5E-5F). Transfection with an empty vector as a negative control had no effect on the response of either SH-SY5Y and IMR5 cells to DS-3032b treatment (Figure 5A-5E). CRISPR-mediated *MDM2* knockout was performed representatively in SH-SY5Y cells, and mimicked the effect of DS-3032b treatment by inhibiting cell viability (Figure 5G-5H). These data highlight the necessity of functional TP53 for DS-3032b activity and indicate that the antiproliferative activity is achieved through reactivating the TP53 signaling.

**Figure 6:**
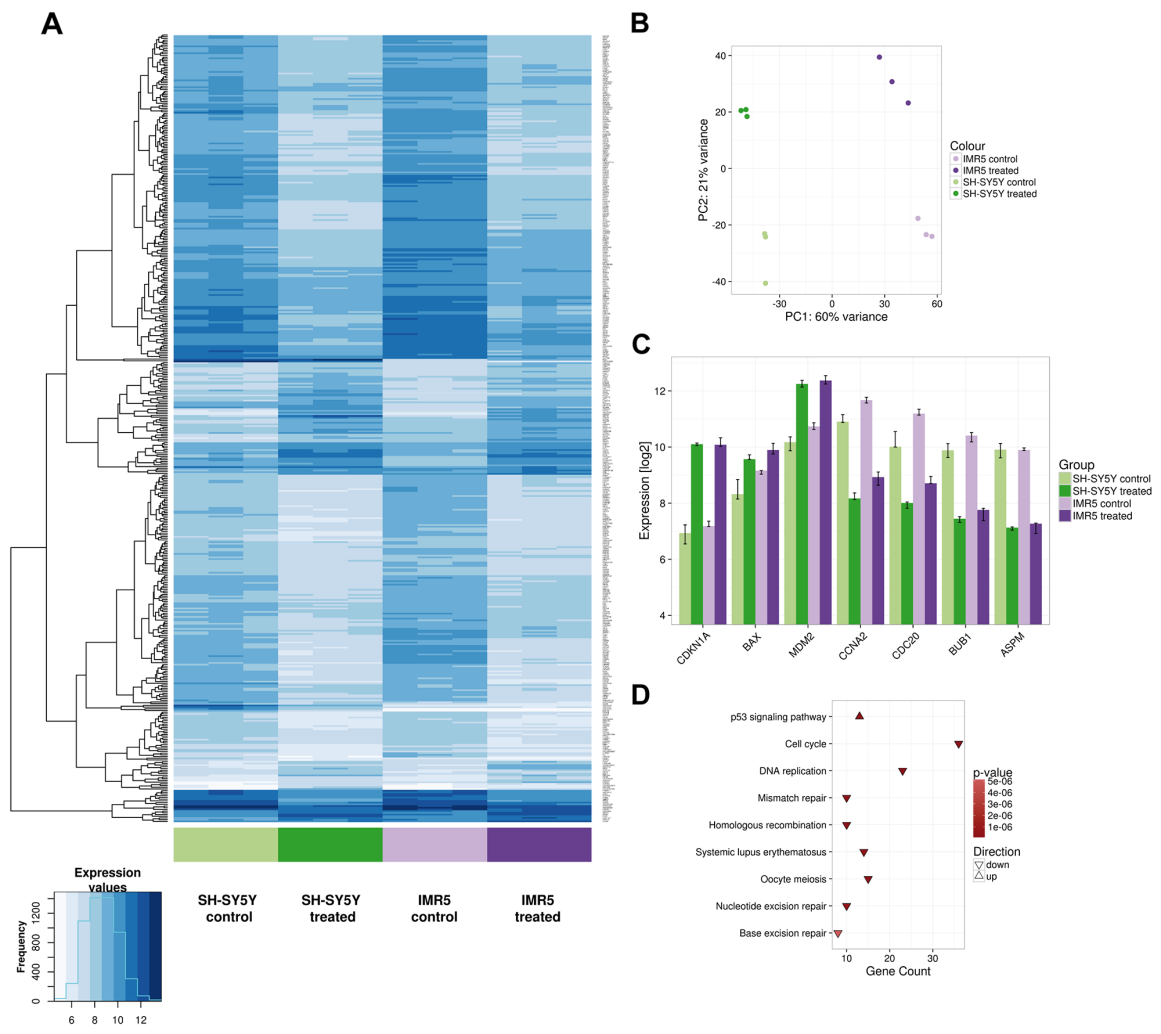
Microarray data confirm TP53 reactivation in neuroblastoma cells, regardless of the presence of a *MYCN* amplification SH-SY5Y and IMR5 cells were treated with 125 nM DS-3032b for 24 h, before harvested for gene expression analysis using an Affymetrix hugene2.0 chip. **(A)** Heatmap visualizes 462 differentially expressed genes after DS-3032b treatment by showing expression values according to the color coding on the bottom left. Colored bars below the heatmap indicate the sample groups. The three replicates of each sample group are shown next to each other. **(B)** Principal component (PC) analysis of the complete data set (24,100 genes, 12 samples) illustrating variation in the expression data between IMR5 and SH-SY5Y cells (PC1), and changes in gene expression induced by treatment with DS-3032b (PC2). **(C)** Changes in expression of *CDKN1A*, *BAX*, *MDM2*, *CCNA2*, *CDC20*, *BUB1* and *ASPM* after DS-3032b treatment. The y-axis is scaled to show the total range of expression values in the complete microarray data set. **(D)** Kyoto Encyclopedia of Genes and Genomes (KEGG) pathways that were found to be over-represented among the differentially expressed genes.

To further support our data on the broad activity of DS-3032b in neuroblastoma cells, we compared gene expression profiles after treatment of SH-SY5Y and IMR5 cells with DS-3032b. Linear model testing identified 462 genes that were significantly differentially expressed after treatment in both cell lines, with 91 genes being up-regulated and 371 genes being down-regulated (Figure 6A). Principal component analysis revealed that 60% of the variation in the expression data was accounted for by the cell line, demonstrating that SH-SY5Y and IMR5 have largely different gene expression profiles (Figure 6B). Treatment with DS-3032b induced pronounced changes in gene expression, accounting for 21% of the variation in the data (Figure 6B). Genes known to be transcriptionally activated by TP53 signaling, such as *CDKN1A*, were prominent among the up-regulated genes (Figure 6C), and gene set enrichment analysis confirmed that TP53 signaling genes were significantly over-represented among the up-regulated genes (Figure 6D). Genes related to cell cycle progression, such as *CCNA2* and *CDC20*, were among genes that were down-regulated by DS-3032b treatment (Figure 6C), and gene set enrichment analysis identified pathways related to cell cycle progression and DNA replication as being over-represented among down-regulated genes (Figure 6D). Our data support a reactivation of TP53 signaling by DS-3032b, regardless of the presence of a *MYCN* amplification.

### DS-3032b delays tumor growth and improves survival in mice xenografted with neuroblastoma cells with functional TP53

**Figure 7:**
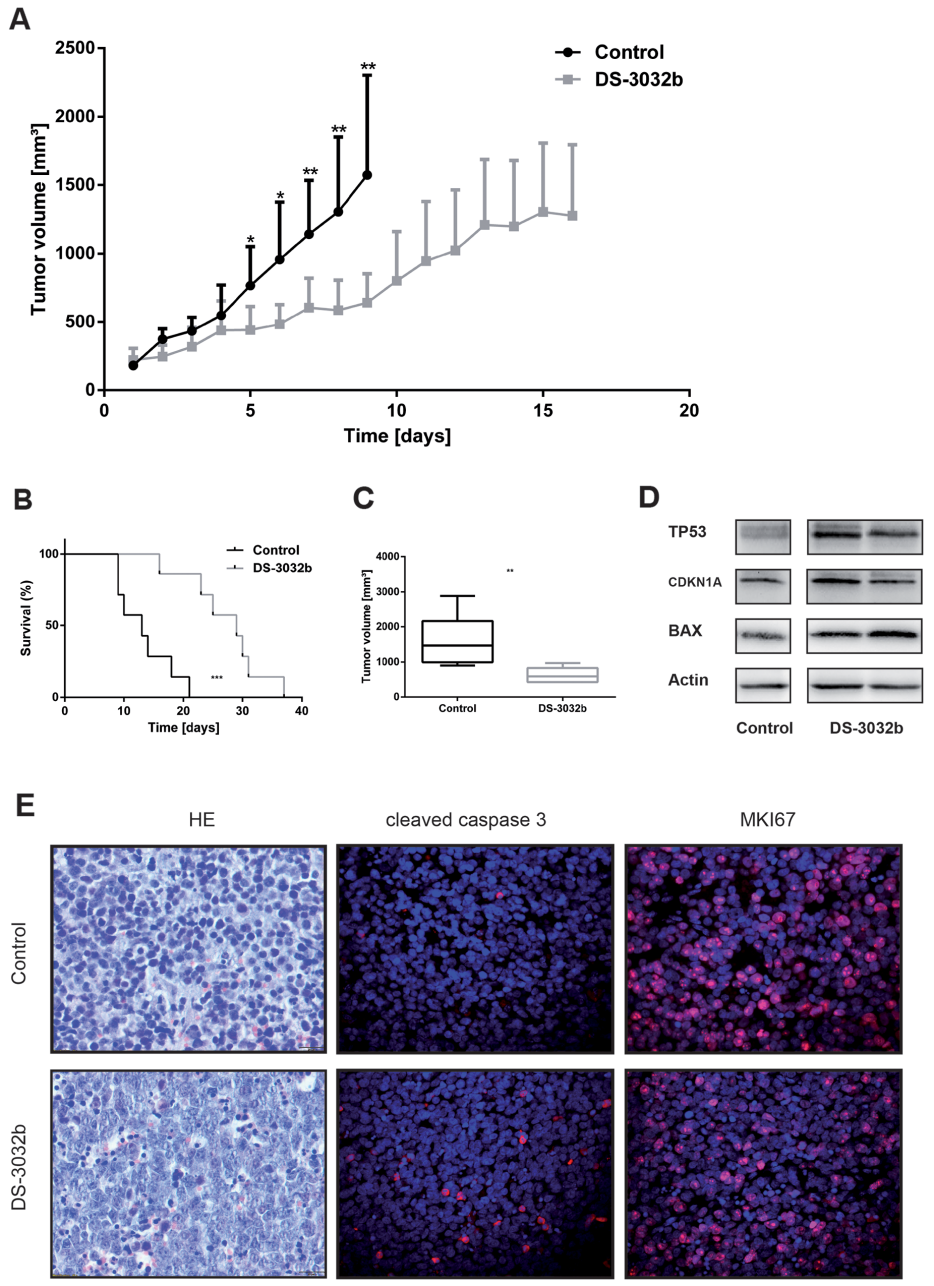
DS-3032b delays tumor growth and improves survival in mice xenografted with wildtype *TP53* neuroblastoma cells **(A)** Tumor growth was significantly delayed in mice treated with DS-3032b (n = 7), compared to controls (n = 7). **(B)** Survival was significantly prolonged by DS-3032b treatment. **(C)** Tumor volume on day 9 of treatment was significantly reduced in mice treated with DS-3032b. **(D)** Expression analysis showed a strong induction of TP53 and a slight induction of CDKN1A and BAX in tumors from 2 mice treated with 4 doses of DS-3032b over a course of 36 h. **(E)** Immunohistochemical analysis showed increased apoptosis (cleaved caspase 3) and decreased proliferation (MKI67) in a representative tumor from mice treated with 4 doses of DS-3032b over a course of 36 h. ^*^ = P < 0.05, ^**^ = P < 0.01, ^***^ = P < 0.001.

We next assessed the efficacy of DS-3032b against SH-SY5Y xenograft tumors in nude mice as an animal model for the potential therapeutic benefit of DS-3032b. Mice with established xenograft tumors were treated for 30 consecutive days with an alternating schedule of 4 days of daily treatment with oral gavages of 50 mg/kg DS-3032b followed by 2 days without treatment. The treatment regimen was well tolerated and did not alter the physical status or behavior of the mice, induce weight loss or produce any other obvious signs of toxicity. Tumor growth was significantly suppressed by DS-3032b treatment in comparison to vehicle control (Figure 7A). Survival in the mouse cohort was significantly prolonged by DS-3032b treatment (Figure 7B). Mean tumor volumes were 640.1 ±80.6 mm^3^ in DS-3032b treated mice compared to 1571.9 ±276.0 mm^3^ in vehicle-treated mice after 9 days of treatment (Figure 7C). DS-3032b treatment activated TP53 signaling, as measured by TP53, CDKNA1 and BAX expression, in the xenograft tumor tissue similarly to the neuroblastoma cell lines grown *in vitro* (Figure 7D). Xenograft tumors from DS-3032b treated mice also displayed a higher proportion of apoptotic cells and fewer proliferating cells as assessed by immunohistochemical detection of cleaved caspase 3 and Ki-67 (MKI67), respectively (Figure 7E). DS-3032b treatment reduced neuroblastoma xenograft tumor growth by activating TP53 signaling *in vivo*.

## DISCUSSION

A prerequisite for the successful use of MDM2 inhibitors is a functional TP53 signaling machinery. As *TP53* mutations occur in less than 2% of primary tumors [2–4], and only in 14% of relapse cases [5], neuroblastoma is expected to be especially susceptible to MDM2 inhibitors. Aberrant activation of the *MDM2* oncogene by gene amplification or inactivation of its inhibitory regulator *CDKN2A* occurs in 36.6% of primary neuroblastomas [5]. Here, we report that high-level *MDM2* expression signals an unfavorable prognosis in a cohort of 476 primary neuroblastomas and correlates with clinical and molecular characteristics of unfavorable tumor biology. Within stage 4 neuroblastoma, correlation between *MDM2* expression and unfavorable prognosis is limited to *MYCN* non-amplified tumors, indicating that the correlation is independent of *MYCN* status. Our results confirm a smaller retrospective study analyzing *MDM2* expression in 91 primary neuroblastomas, in which *MDM2* expression negatively correlated with event-free survival after three years [32]. However, a recent study analyzing *MDM2* promotor SNP309 in 496 neuroblastoma patients, a single nucleotide polymorphism in the *MDM2* promotor that was shown to increase *MDM2* expression [33], did not find an association between this polymorphism and overall or event-free survival [34]. There is clinical evidence that TP53 signaling contributes to tumor activity in neuroblastomas, but it is too early to draw any conclusions.

Here, we present the preclinical evaluation of the novel small-molecule MDM2 inhibitor, DS-3032b, for its value for treating patients with high-risk, refractory or relapsed neuroblastoma. Treatment with DS-3032b stabilized TP53 and selectively induced CDKNA1, BAX and MDM2 expression in neuroblastoma cells with wildtype *TP53*. TP53 accumulation and subsequent TP53 target gene activation have also been demonstrated in neuroblastoma cells with wildtype *TP53* following treatment with other MDM2 inhibitors, including RG7112, RG7388, MI-63, NDD0005 and MI-773 [18–23]. Here, we performed expression profiling of neuroblastoma cells with and without DS-3032b treatment in order to further scrutinize functional mechanisms of DS-3032b. Gene set enrichment analysis using Kyoto Encyclopedia of Genes and Genomes (KEGG) pathways revealed a significant induction of genes involved in TP53-dependent cell cycle arrest and apoptosis. Interestingly, we could not identify relevant off-target effects possibly due to high specificity of DS-3032b. DS-3032b effectively inhibited cell viability, proliferation and migration by inducing apoptosis, G1 cell cycle arrest and senescence in the work we present here. All neuroblastoma cell lines with wildtype *TP53* responded to DS-3032b treatment, regardless of the presence of *MYCN* amplifications, indicating that this MDM2 inhibitor may benefit high-risk patients with neuroblastomas having several molecular backgrounds. Independence of *MYCN* status on *in vitro* potency may be a particular feature of DS-3032b among MDM2 inhibitors. Nutlin-3 and MI-63, both MDM2 inhibitors, have more effectively suppressed proliferation of *MYCN*-amplified neuroblastoma cells compared to cells lacking *MYCN* amplifications [35]. We characterized the strong pro-apoptotic activity induced by DS-3032b in flow cytometry experiments with neuroblastoma cells, and demonstrated that DS-3032b induces apoptosis in a primary neuroblastoma cell line. Functional outcome of DS-3032b treatment was primarily dependent on the *TP53* status of the cell line, as evidenced by the finding that stable, ectopic expression of dn-TP53 attenuated antitumor activity of DS-3032b in SH-SY5Y and IMR5 cells. The remaining antiproliferative and pro-apoptotic activity of DS-3032b in cells with dn-TP53 could be mediated by TP73, a TP53 homolog also negatively regulated by MDM2 [36]. Lau et al. have reported that Nutlin-3 treatment results in TP73-dependent apoptosis [37]. DS-3032b may inhibit TP73-MDM2 binding in an analogous manner. The novel MDM2 antagonist DS-3032b was shown to be effective in neuroblastoma cells with wildtype *TP53* by reactivating TP53 signaling.

Although we show that DS-3032b has potent antitumor activity as a single agent, treatment using MDM2 antagonists as single agents is likely to spur development of resistance mechanisms [38] and be unwise for patients. Patients with high-risk neuroblastoma are currently treated with genotoxic chemotherapy and/or radiotherapy. Tumor cells respond to DNA damage induced by chemotherapy or radiotherapy by activating machinery that halts cell cycle progression and stimulates DNA repair, or if the damage is too great, inducing senescence or apoptosis [39]. Chen et al. have demonstrated that the MDM2 antagonist RG7388 works synergistically with chemotherapeutic agents used in neuroblastoma treatment (cisplatin, doxorubicin, topotecan, temozolomide and busulfan) to achieve a stronger apoptosis induction in neuroblastoma cells *in vitro* [19]. Integrating DS-3032b treatment in the high-risk treatment protocol may be able to release the brakes on this machinery and increase apoptosis in neuroblastoma cells induced by genotoxic chemotherapy and/or radiotherapy.

We demonstrate that DS-3032b inhibits proliferation and increases apoptosis in neuroblastoma xenografts in nude mice. Nutlin-3, RG7112 and RG7388 have also been demonstrated to have antitumor activity against neuroblastoma mouse models [15, 18, 22]. To date, however, none of the MDM2 inhibitors has entered clinical treatment of patients with neuroblastoma. RG7112, the first small-molecule MDM2 antagonist in clinical testing, demonstrated significant gastrointestinal and hematopoietic side effects but only modest clinical activity in phase I clinical trials conducted in adult patients with liposarcoma or leukemia [40, 41]. No visible signs of toxicity were observed in mice treated with DS-3032b in the work presented here. Preliminary results from an ongoing phase 1 trial treating adult patients with relapsed or refractory acute myeloid leukemia or high-risk myelodysplastic syndrome with DS-3032b as a single agent have demonstrated acceptable clinical side effects and efficacy, with reduction of bone marrow blasts by the end of one cycle in 57.7% of patients [30]. The indications from both our preclinical animal experiments and initial data from trials in adult patients supports the view that DS-3032b achieves MDM2 inhibition with fewer toxic side effects, and is likely a promising MDM2 small molecule inhibitor for entry into pediatric patient trials for neuroblastoma.

Our preclinical data adds further evidence that targeted disruption of TP53-MDM2 binding results in potent antitumor activity in neuroblastoma cells. We provide the first preclinical feasibility study of the novel orally available MDM2 antagonist DS-3032b in neuroblastoma. Future preclinical studies with DS-3032b will be needed to determine the optimal dosage and timing for combination with established chemo- and radiotherapy treatment schedules. The pleiotropic activities and low toxicity suggest DS-3032b as the best available candidate to inhibit MDM2 for integration into clinical treatment protocols for high-risk neuroblastoma.

## MATERIALS AND METHODS

### Tumor expression re-analysis from existing data

*MDM2* expression values were derived from existing whole-genome expression profiles from 649 primary neuroblastoma samples [31], 173 samples lacked survival data and had to be omitted from the survival analysis. Kaplan–Meier analyses for the neuroblastoma cohort were performed online in the R2 platform (http://r2.amc.nl) and the resulting survival curve and P values (log-rank test) were downloaded. Tumor samples were classified into high- or low-expressing groups according to whether *MDM2* expression was greater or lower than the third quartile *MDM2* expression.

### Cell culture

The human neuroblastoma cell lines Kelly, SK-N-SH, SH-SY5Y, IMR32, IMR5 and LAN5 were grown in RPMI 1640 supplemented with 10% FCS, L-glutamine and 1% penicillin/streptomycin. All cell lines were authenticated by short tandem genotyping by the German Collection of Microorganisms and Cell cultures (Braunschweig, Germany) prior to experiments. *TP53* mutational status of all cell lines was analyzed using Sanger sequencing and multiplex ligation-dependent probe amplification in the laboratories of Reinhard Schneppenheim at the University Hospital Hamburg, Germany. DS-3032b (Daiichi Sankyo) was dissolved in DMSO and stored as a 20 mM stock solution in small aliquots at -20°C. Cells were exposed to 0 – 2000 nM DS-3032b, prepared as serial dilutions in complete culture medium, for the period indicated. The final DMSO concentration was kept at or below 0.01%. For caspase 3 activity experiments, OHC-NB-1 (wildtype *TP53*) was used as a primary neuroblastoma cell line.

For rescue experiments, IMR5 and SH-SY5Y cells (wildtype *TP53*) were transfected with a pMSCVpuro plasmid encoding dn-TP53 mutant (donated by Martin Eilers) containing the Val135 *TP53* mutation or the pMSCVpuro plasmid alone as a control using TurboFect (Thermo Fisher Scientific). Transduced cells were selected by addition of puromycin (0.5 μg/ml) to the medium for 7 days.

For CRISPR-mediated *MDM2* knockout, guide RNA (gRNA) sequence was designed using the Zhang lab CRISPR design tool (http://crispr.mit.edu) and the Broad Institute sgRNA designer (http://www.broadinstitute.org/rnai/public/analysis-tools/sgrna-design). Global *MDM2* knockout was achieved by two gRNAs targeting exon 2 of *MDM2* (sequence 5’ -> 3’: GTGGTTACAGCACCATCAGT and AGCTTCGGAACAAGAGACCC). Upon selection of gRNA, oligonucleotides were ordered, annealed and ligated into BbsI-digested CRISPR vectors px458 (pSpCas9(BB)-2A-GFP; donated by Feng Zhang (Addgene plasmid #48138)) and px459 (pSpCas9(BB)-2A-Puro V2.0; donated by Feng Zhang (Addgene plasmid #62988)). Generated vectors were validated by sequencing and directly used for cell-transfection using the Gene Pulser Xcell™ Electroporation system (Bio-Rad Laboratories) applying the settings suggested for neuroblastoma cell lines (200 V, 20 ms, 1 pulse). Fluorescence activated cell sorting for green fluorescent protein (GFP)-expressing cells was done 24 h after electroporation. Cell viability was measured 24, 48 and 72 h after sorting, and cells were harvested for western blot analysis 7 days after sorting.

### Real-time quantitative reverse transcription-PCR

Total RNA was isolated from cells with use of the NucleoSpin kit (Machenery-Nagel) and cDNA synthesis was performed using the SuperScript III reverse transcription kit (Invitrogen, Thermo Fisher Scientific). *MDM2* expression was monitored using real-time polymerase chain reaction (PCR) using Assays on Demand (Applied Biosystems). Expression values were normalized to *GAPDH* (Applied Biosystems).

### Western blot analysis

Cells treated with 0, 125 and 250 nM DS-3032b for 48 h were solubilized in lysis buffer containing 50 mM Tris-HCl, 1% NP-40, 0.25% sodium deoxycholate, 150 nM NaCl, and 1 mM EGTA. Total protein (30-50 μg) was fractionated on 10–12% sodium dodecyl sulfate polyacrylamide gel, transferred to a nitrocellulose membrane (Bio-Rad Laboratories) or polyvinylidene difluoride membrane (Roche). The following primary antibodies were used: MDM2 (1:500, Santa Cruz Biotechnology), TP53 (1:500, Santa Cruz Biotechnology), CDKN1A (1:500, Santa Cruz Biotechnology), BAX (1:1000, Cell Signaling Technology) and Actin (1:7500, Sigma-Aldrich) as the loading control. Anti-rabbit horseradish peroxidase (HRP, 1:5000, GE Healthcare) and anti-mouse HRP (1:5000, GE Healthcare) were used as secondary antibodies. Proteins were visualized using enhanced chemiluminescence detection reagents (Thermo Fisher Scientific) and analyzed on a Fusion FX7 detection device (Peqlab, Erlangen, Germany).

### Cell viability, proliferation and senescence analysis

Cells were seeded in triplicate onto 96-well plates, incubated for 24 h to permit adherence, then treated with 0 - 2000 nM DS-3032b for 24, 48 or 72 h. Cell viability was determined using the 2,3-Bis(2-methoxy-4-nitro-5-sulfophenyl)-2H-tetrazolium-5-carboxanilide (XTT) assay (Sigma-Aldrich) according to the manufacturer's protocol. The IC50 was calculated using GraphPad Prism 6.0 (GraphPad Software). Cell proliferation was determined using the BrdU ELISA assay (Roche) according to the manufacturer's protocol. Senescence was measured using the fluorometric SA-β-gal activity assay (Cell Biolabs Inc.) according to the manufacturer's protocol and corrected for cell viability (XTT assay).

### Migration analysis

A modified scratch assay was used for analysis of cell migration [42]. To minimize cell proliferation, tumor cells were starved in 0.5% serum medium, cultured in 12-well plates, and scratched with the fine end of 100 μl pipette tips (time 0). Plates were washed with phosphate buffered saline (PBS) to remove detached cells, and incubated with 0 and 250 nM DS-3032b for 24 h. The scratched area was photographed using 10x objective and the open image area was calculated using the software TScratch [43].

### Cell cycle and apoptosis analysis

Cells were seeded in triplicate in 6-well plates, incubated for 24 h to permit adherence, and treated with 0, 125 and 250 nM DS-3032b for 48 h. Prior to cell cycle analysis, cells were washed with PBS, resuspended in 450 μl PBS with 50 μg/ml propidium iodide (PI, Sigma-Aldrich) and 100 μg/ml RNAse (Sigma-Aldrich), and incubated at room temperature for at least 10 min. Prior to apoptosis analysis, cells were resuspended in 100 μl binding buffer (10 mM Hepes, 140 mM NaCl, 2.5 mM CaCl_2_) with 2.5 μl fluorescein isothiocyanite (FITC)-conjugated antibodies against annexin V (BD Biosciences) and 4 μl PI (50ug/ml), and incubated at 4°C for 15 min. 10^5^ cells were analyzed on the LSRFortessa X-20 using FACSDiva™ software (BD Biosciences). Data were analyzed using FlowJo v10 (FlowJo).

### Caspase 3 activity analysis

Cells were seeded in 100 mm plates, incubated for 24 h to permit adherence, and then treated with 0, 125 and 250 nM DS-3032b for 48 h. After cell lysis in Cell Lysis Buffer (BioVision), 50 μg protein were incubated with Ac-DEVD-AFC (Biomol) at 37°C for 1 h, and caspase 3 activity was measured with a multi-well fluorescence plate reader.

### Affymetrix microarray analysis

SH-SY5Y and IMR5 cells plated at 10^6^ cells/well in 6-well plates, then after 24 h for attachment, were treated in triplicate with control medium and medium containing 125 nM DS-3032b for 24 h. Gene expression was assayed using the Affymetrix GeneChip Human Gene 2.0 ST Array. Microarray data were processed with R/Bioconductor version 3.3 using the Brainarray annotation for hugene2.0 and the vsn normalization method [44]. Differentially expressed genes after treatment were derived using the limma package [45]. Linear model testing was applied to test whether the treatment effect was significantly different from 0. Test P values were corrected for multiple testing using the False Discovery Rate method. Genes with a corrected P value < 0.1 and an absolute fold-change greater than 2 were called significantly differentially expressed. A heatmap of differentially expressed genes was created using the gplots package. Euclidean distance and complete-linkage clustering were used to cluster genes (rows), samples (columns) were not clustered. The Bioconductor package clusterProfiler was used to identify KEGG pathways that were significantly often associated to genes in a given gene list. Pathways with a P value < 0.0001 were called significantly over-represented.

### Xenograft tumor experiments in nude mice

SH-SY5Y cells were cultured to 80% confluency, harvested and suspended in a 200 μl volume of PBS:Matrigel (BD Biosciences) for subcutaneous inoculation (2 × 10^7^ cells per mouse, n =14 mice) into the right flank of 4-week-old female athymic NCr (nu/nu) mice. Mice were randomly assigned to either DS-3032b or vehicle control groups (n = 7 mice per group) after tumors reached 150–250 mm^3^ in size. DS-3032b or vehicle control was administered by oral gavage at a dose of 50 mg per kg body weight in 0.5% methylcellulose solution (400 cps, Sigma-Aldrich). The treatment was carried out for 30 consecutive days with an alternating schedule of 4 days of daily treatment followed by 2 days without treatment. Tumor growth was monitored using a caliper, and tumor volume was calculated using the formula (breadth × length × height)/2. Mice were euthanized by cervical dislocation when tumor size exceeded 2000 mm^3^. To examine the effects of DS-3032b treatment on reactivation of the TP53 signaling, nu/nu mice with established SH-SY5Y xenografts were treated orally with 4 doses of DS-3032b (100 mg per kg body weight) over a course of 36 h (doses were given at 0, 12, 24 and 36 h; n = 2). Mice were euthanized by cervical dislocation 4 h after the last dose. The tumor was removed, and half of each tissue was snap-frozen in liquid nitrogen then stored at -80°C, and the other half of tissue was formalin fixed and paraffin embedded. All animal experiments were performed in accordance with the Council of Europe guidelines for accommodation and care of laboratory animals and protocols were approved by the Institutional Ethical Commission for Animal Experimentation.

### Xenograft tumor immunohistochemistry

Paraffin-embedded 5 μm sections of SH-SY5Y xenografts from mice that had been treated with 4 oral doses of 100 mg/kg DS-3032b or vehicle control for 36 h were deparaffinized in xylene, followed by rehydration by transfer through a graded alcohol series. The following primary antibodies were used: MKI67 (rabbit anti-human, 1:100, Cell Signaling Technology) and cleaved caspase 3 (rabbit anti-human, 1:100, Cell Signaling Technology). Sections were incubated with primary antibody at 4°C overnight. An Alexa594-conjugated secondary antibody (donkey anti-rabbit, 1:350, Invitrogen, Thermo Fisher Scientific) was applied for 45 min at room temperature. After counterstaining of the nucleus with 4’,6-diamidino-2-phenylindole (DAPI, 1:1000, Roche) the slides were mounted using Vectashield anti-fade mounting medium (Vector Laboratories). Images were recorded on a high-resolution fluorescence microscope (Zeiss Axiovert 200).

### Statistical analysis

GraphPad Prism 6.0 was used for graphical presentation, calculation of standard deviation and further statistical analysis. Statistical significance of differences between treatment groups was determined using a Student's *t* test. For *in vivo* experiments Kaplan-Meier survival curves were applied and statistical analysis was performed using the log-rank test.

## SUPPLEMENTARY MATERIALS FIGURES AND TABLES



## Abbreviations

CRISPR: clustered regularly interspaced short palindromic repeats
INSS: International Neuroblastoma Staging System
