# Supplementary material for: Reactivating TP53 signaling by the novel MDM2 inhibitor DS-3032b as a therapeutic option for high-risk neuroblastoma
Source: Oncotarget. 2017 Dec 18;9(2):2304–19. doi: 10.18632/oncotarget.23409 (PMC5788641; doi:10.18632/oncotarget.23409)
Supplement: Supplementary file 1 [file oncotarget-09-2304-s001.pdf]

# Reactivating TP53 signaling by the novel MDM2 inhibitor DS-3032b as a therapeutic option for high-risk neuroblastoma

## SUPPLEMENTARY MATERIALS

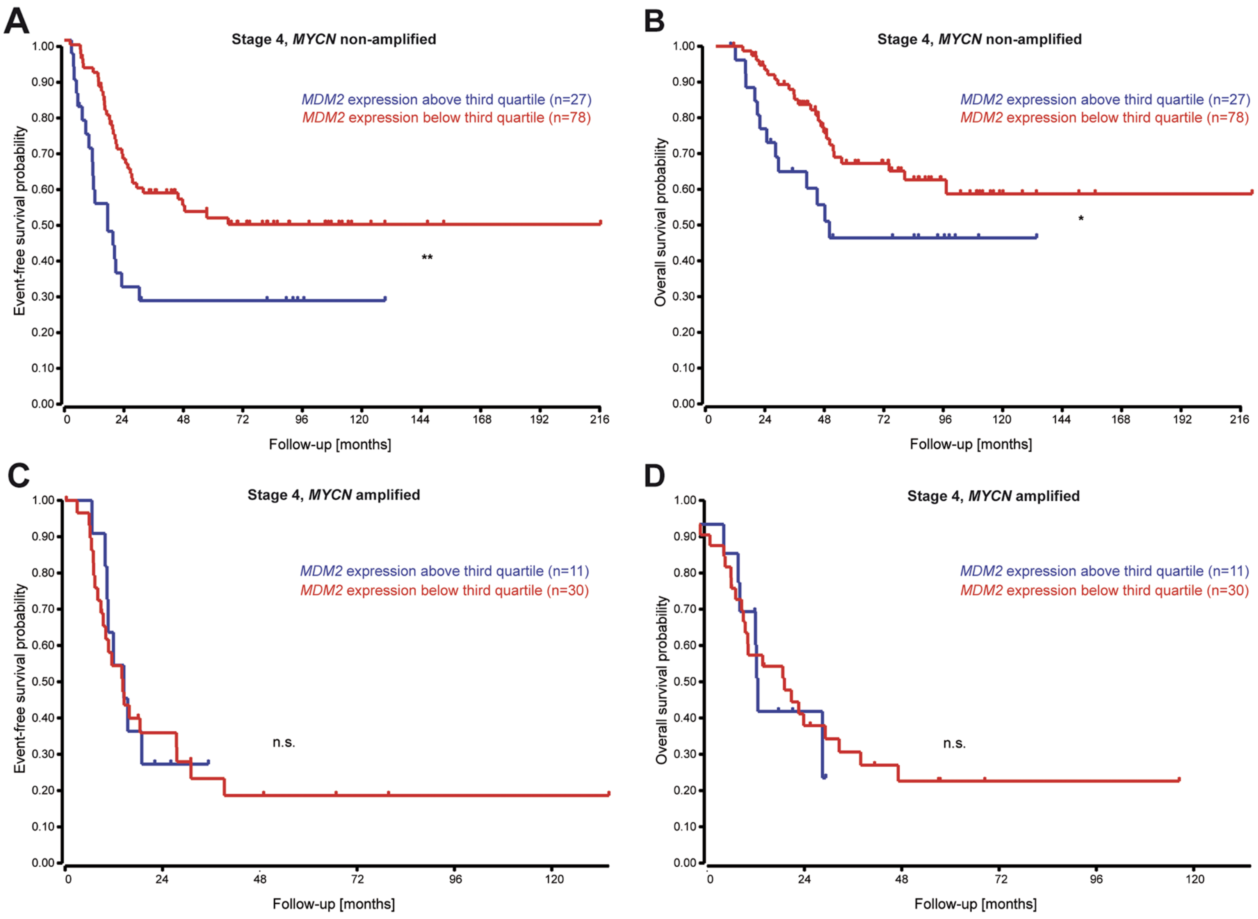


**Supplementary Figure 1: Elevated *MDM2* expression levels in tumors are associated with poor patient overall and event-free survival in stage 4, *MYCN* non-amplified neuroblastoma.** Tumors from a cohort of 476 primary tumors were classified into high- or low-expressing groups according to whether *MDM2* expression was greater or lower than the third quartile *MDM2* expression. Kaplan–Meier analysis of overall and event-free patient survival for **(A-B)** stage 4, *MYCN* non-amplified tumors and **(C-D)** stage 4, *MYCN* amplified tumors. $^{*}$ = $P < 0.05$, $^{**}$ = $P < 0.01$, n. s. = $P \geq 0.05$.
